# Spatial constraints on chromosomes are instrumental to meiotic pairing

**DOI:** 10.1242/jcs.253724

**Published:** 2020-11-30

**Authors:** Miao Tian, Christiane Agreiter, Josef Loidl

**Affiliations:** Department of Chromosome Biology, Max Perutz Laboratories, University of Vienna, A-1030 Vienna, Austria

**Keywords:** Meiosis, Chromosome pairing, Centromere, Telomere

## Abstract

In most eukaryotes, the meiotic chromosomal bouquet (comprising clustered chromosome ends) provides an ordered chromosome arrangement that facilitates pairing and recombination between homologous chromosomes. In the protist *Tetrahymena thermophila*, the meiotic prophase nucleus stretches enormously, and chromosomes assume a bouquet-like arrangement in which telomeres and centromeres are attached to opposite poles of the nucleus. We have identified and characterized three meiosis-specific genes [meiotic nuclear elongation 1-3 (*MELG1-3*)] that control nuclear elongation, and centromere and telomere clustering. The Melg proteins interact with cytoskeletal and telomere-associated proteins, and probably repurpose them for reorganizing the meiotic prophase nucleus. A lack of sequence similarity between the *Tetrahymena* proteins responsible for telomere clustering and bouquet proteins of other organisms suggests that the *Tetrahymena* bouquet is analogous, rather than homologous, to the conserved eukaryotic bouquet. We also report that centromere clustering is more important than telomere clustering for homologous pairing. Therefore, we speculate that centromere clustering may have been the primordial mechanism for chromosome pairing in early eukaryotes.

## Abstract (precis)

**Summary:** Mutagenic and cytological analyses allow the characterization of proteins that are crucial for the bouquet-like chromosome arrangement in *Tetrahymena* and propose that centromere clustering is important for pairing.

## INTRODUCTION

The issue of how homologous chromosomes move and find each other during meiotic prophase is much debated. Membrane tethering of telomeres, centromeres or other specialized chromosome regions, together with movements driven by cytoskeletal elements, enables chromosomes to find and pair with their homologous partners. Although the bouquet arrangement with telomeres clustered at the nuclear periphery is prevalent in most eukaryotes (Scherthan, 2001; Zickler and Kleckner, 2016), additional or alternative mechanisms for aligning chromosomes do exist (see Da Ines and White, 2015; Loidl, 2016).

**Fig. 1.**
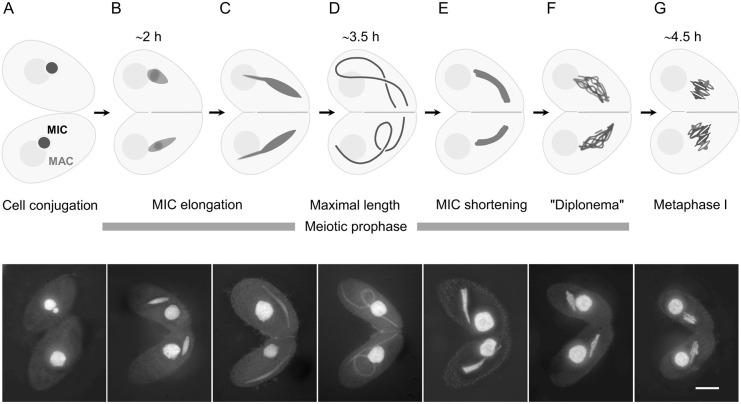
**Schematic diagram and DAPI-stained wild-type cells in meiosis.** (A) Starved cells before mating. The germline nucleus (micronucleus, MIC) resides within a pocket of the polyploid somatic nucleus (macronucleus, MAC). (B) Once cells have mated, the germline nuclei synchronously initiate meiosis. They detach from the MACs and start to elongate. (C) Germline nuclei in the process of elongation. (D) Cell pair with fully elongated germline nuclei. (E) Germline nuclei in the process of shortening. (F) Chromatin condenses in the germline nucleus at a stage corresponding to diplonema/diakinesis. (G) During metaphase I, condensed bivalents are arranged in a metaphase plate. Approximate time in hours (h) after induction of cell mating is indicated. Scale bar: 10 µm.

Meiotic prophase nuclei of the unicellular protist *Tetrahymena thermophila* undergo a remarkable reorganization – they stretch to approximately twice the length of the cell (Sugai and Hiwatashi, 1974; Fig. 1). Within the elongated nucleus, chromosome arms are arranged side by side, with centromeres and telomeres attached to opposite tips (Loidl et al., 2012). The polarized orientation of chromosome arms resembles the bouquet. Nuclear elongation depends on Spo11-induced DNA double-strand breaks (DSBs) (Mochizuki et al., 2008; Tian and Loidl, 2018) and DSB sensing by the Atr1 kinase (Loidl and Mochizuki, 2009), and is driven by intranuclear microtubules (MTs) (Davidson and LaFountain, 1975; Kaczanowski et al., 1985; Loidl and Mochizuki, 2009; Wolfe et al., 1976).

It is unclear how the nuclear MT apparatus becomes reorganized to push apart the opposite ends of the nucleus, and how centromeres and telomeres become linked to MTs or the nuclear envelope in order to span the poles of the elongated nucleus. For recruitment to the tip of the elongated nucleus, centromeres must possess functional centromere-specific histone Cna1 (Loidl et al., 2012). However, the factors responsible for tethering both centromeres and telomeres to opposing ends of the nucleus are unknown. We have identified three genes (*MELG1-3*) required for prophase nuclear organization, and studied the roles of the individual Melg proteins in this process.

Nuclear elongation begins ∼2 h after the induction of meiosis, and molecular studies have revealed that crossing over is initiated during the elongated state and is completed ∼2 h later when nuclei begin to shorten (Loidl et al., 2012). Although it is reasonable to assume that the particular style of nuclear reorganization in *Tetrahymena* promotes homologous pairing and recombination (Loidl et al., 2012; Loidl and Scherthan, 2004), it has not been possible to directly prove this because mutants that are defective in elongation (such as *spo11*Δ) do not initiate recombination. Moreover, blocking elongation by chemical inhibition of Atr1 or MT formation results in meiotic arrest, which precludes bivalent formation. In contrast, in knockout mutants of the newly identified genes, nuclear elongation and telomere and/or centromere clustering was impaired, but subsequent steps in meiosis were executed. This phenotype enabled us to confirm that bivalent formation depends on nuclear reorganization.

## RESULTS

### Melg proteins promote meiotic nuclear elongation

**Fig. 2.**
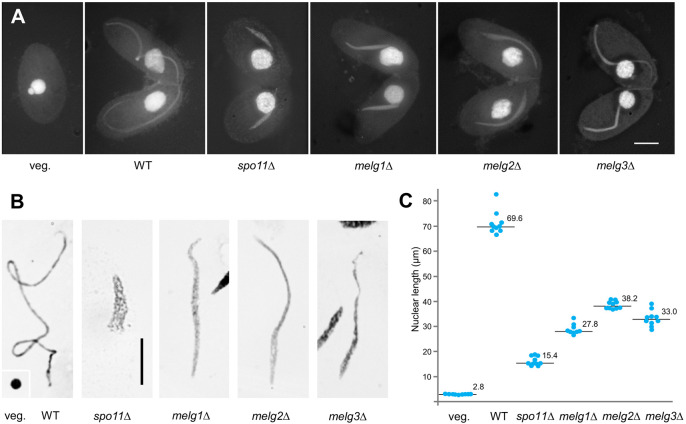
**Elongation of meiotic prophase nuclei.** (A) DAPI staining of a vegetative cell (veg.) and mating cell pairs at the stage of maximal nuclear elongation. (B) Giemsa-stained representative nuclei of isolated WT and mutant nuclei. Inset in the leftmost panel shows a non-meiotic (veg.) germline nucleus. (C) Scatter diagram showing the ten longest nuclei of each genotype. Horizontal lines represent the median values. Standard deviations are not given because lengths do not represent average values but were biased toward the longest observed (see main text). Diameters of non-meiotic germline nuclei and lengths of *spo11*Δ nuclei are included for comparison. Scale bars: 10 µm.

The *Tetrahymena* Functional Genomics Database (TetraFGD, http://tfgd.ihb.ac.cn/; Xiong et al., 2013) was screened for genes with peak transcription at an early stage of conjugation (corresponding to meiotic prophase). Among the over 80 candidate meiosis genes that were knocked out, we identified three genes that are essential for the full elongation of prophase nuclei (Fig. 2A-C; Fig. S1). We named these genes *MELG1*, *MELG2* and *MELG3* (for meiotic nuclear elongation), and the corresponding proteins Melg1, Melg2 and Melg3.

In each mutant, nuclear elongation was quantified by measuring at least 30 of the most elongated meiotic nuclei, as identified by visual inspection. Maximal elongation was determined as the median length of the ten longest nuclei. It was 27.8 µm for *melg1*Δ, 38.2 µm for *melg2*Δ, and 33.0 µm for *melg3*Δ, versus 69.6 µm in the wild type (WT) (Fig. 2C). Notably, elongation was not completely abolished even in the absence of Spo11, possibly because the initial formation of intranuclear MTs is DSB independent.

As impaired nuclear elongation is characteristic of defective DSB formation (such as in the absence of *SPO11* or *PARS11*) or DSB sensing (such as in the absence of *ATR1*) (Loidl and Mochizuki, 2009; Mochizuki et al., 2008; Tian and Loidl, 2018; Fig. 2), DSB formation and processing was tested in the *melg* mutants. Foci of the meiosis-specific recombination protein Dmc1 (which is known to localize to DSBs; Howard-Till et al., 2011) were present in all three mutants (Fig. S1B). In addition, unlike in the *spo11*Δ mutant, normal elongation could not be restored by UV-induced DNA damage in any of the *melg* mutants (Fig. S1C). These observations indicate that DSBs were formed and sensed, and suggest that the elongation defect has mechanistic causes.

### Melg proteins are important for centromere and telomere clustering

**Fig. 3.**
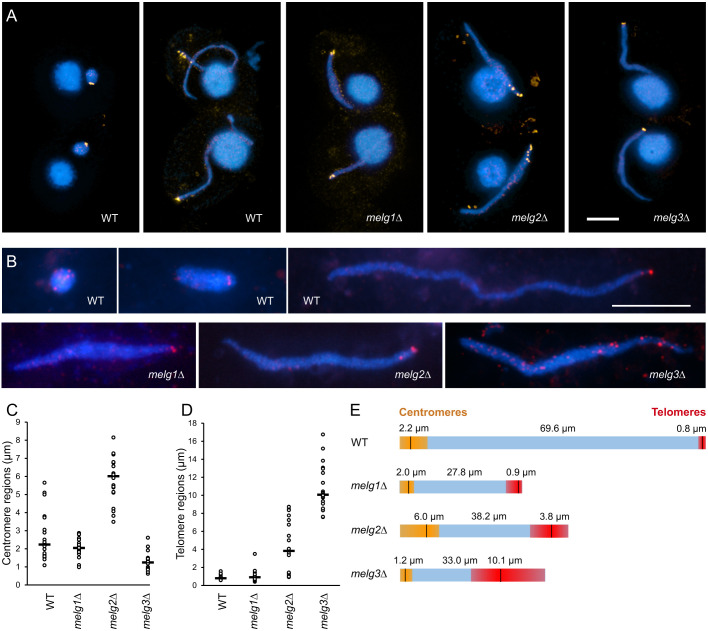
**Centromere and telomere distribution within meiotic nuclei.** (A) Centromeres (Cna1, orange) in the WT before elongation and during full elongation, and in *melg* mutants. (B) Telomeres (telFISH, red) in elongating and fully elongated nuclei of the WT and in the mutants. (C) Lengths of regions occupied by centromere signals, as measured in 20 nuclei (bar, median length). (D) Lengths of regions occupied by telomere signals, as measured in 20 nuclei (bar, median length). (E) Diagrams showing median nuclear lengths (from Fig. 2C) and regions occupied by centromeres (orange) and telomeres (red). Vertical lines indicate the median lengths of the regions (from C and D). Ratios of nuclear lengths to centromere- and telomere-occupied regions within genotypes are not proportional because they were measured following different fixation methods, but the differences in nuclear lengths and centromere- and telomere-occupied regions between the genotypes are represented correctly. Scale bars: 10 µm.

We next studied the localization of centromeres and telomeres because both centromere and telomere attachment are needed for chromosome arms to span across the elongated nucleus and adopt parallel alignment (Loidl et al., 2012). Centromeres were marked with an antibody against the centromeric histone Cna1 and telomeres were marked by fluorescence *in situ* hybridization (FISH) using a probe against the telomere repeat sequence (Fig. 3). In the *melg1*Δ mutant, centromeres and telomeres were in tight clusters at opposite tips of the nucleus. In the *melg2*Δ mutant, both centromeres and telomeres formed more dispersed clusters at the nuclear tips, and in the *melg3*Δ mutant, centromeres were clustered at the nuclear tip, whereas telomeres were dispersed along half of the length of the nucleus (Fig. 3).

### Pairing and bivalent formation are differentially affected in *melg* mutants

**Fig. 4.**
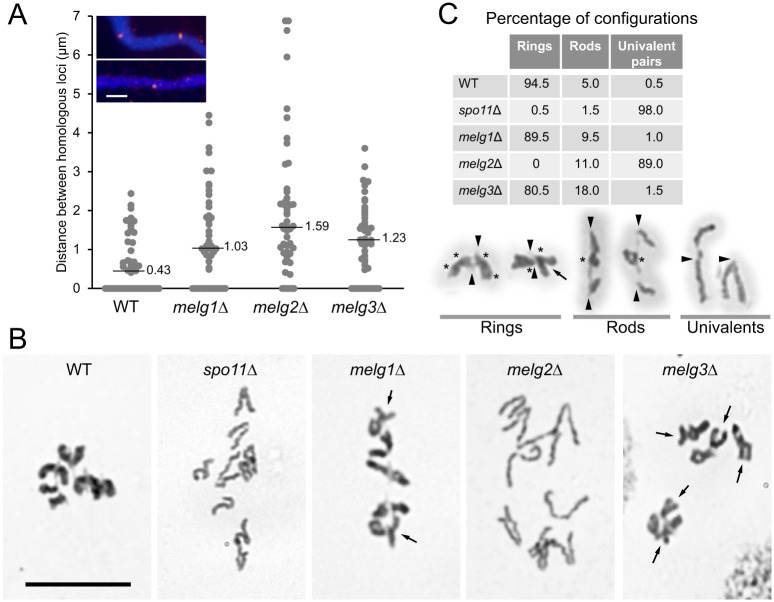
**Pairing and bivalent formation.** (A) Quantification of pairing. Distances between centres of FISH signals are given in microns. A total of 50 nuclei were evaluated for each genotype. Horizontal lines indicate median values. Inset: examples of unpaired and paired FISH signals. Scale bar: 2 µm. (B) Giemsa-stained isolated diakinesis/metaphase I nuclei of *melg1-3* mutants together with WT and *spo11*Δ controls. In *melg1*Δ and *melg3*Δ, bivalent ends are often splayed (arrows). Scale bar: 10 µm. (C) Quantification of bivalent formation (*n*=200) and examples of chromosome configurations. Arrowheads denote centromeres, and asterisks denote possible chiasmata. X-shaped bivalents are topological rings as they are connected on both sides of the centromere, but splayed ends (arrows in B,C) suggest a reduced number of chiasmata compared with WT.

We next studied how defective prophase chromosome arrangements affect homologous pairing (quantified by measuring the distance between FISH-labelled homologous loci) and bivalent formation in diakinesis to metaphase I. Pairing was most impaired in the *melg2*Δ mutant (Fig. 4A) and, accordingly, bivalent formation was almost completely abolished. In contrast, pairing and bivalent formation were only mildly impaired in both *melg1*Δ and *melg3*Δ (Fig. 4B,C). From the frequencies of ring and rod bivalents and univalents, we estimated the number of crossovers (COs) by assuming that COs were randomly assigned to the ten arms of the five chromosome pairs (for formulas, see Table S1). The average numbers per cell were 37 COs for the WT and 27, 0.5 and 22 COs, for *melg1*Δ, *melg2*Δ and *melg*3Δ, respectively. Thus, *melg1*Δ and *melg*3Δ had moderate reductions in CO and, accordingly, meiotic divisions progressed normally (Fig. S1). In contrast, meiotic divisions were abnormal in *melg2*Δ (Figs S1,S2). Evidence from *spo11*Δ shows that, apart from nondisjunction, the absence of COs does not necessarily affect meiotic divisions (Mochizuki et al., 2008). However, *melg2*Δ had problems with delayed or incomplete chromosome separation in anaphase I, along with chromosome loss and the production of vestigial nuclei in anaphase II (Figs S1,S2). These defects are consistent with a possible function for the Melg2 protein in MT stabilization (see below). Pairing and bivalent formation were most affected in the *melg2*Δ mutant, whereas they were least affected in the *melg1*Δ mutant and mildly affected in the *melg3*Δ mutant. This finding indicates that centromere attachment (which is preserved in *melg1*Δ and *melg3*Δ) is sufficient to achieve a substantial degree of homologous chromosome pre-alignment.

### Localization and interactions of Melg proteins

**Fig. 5.**
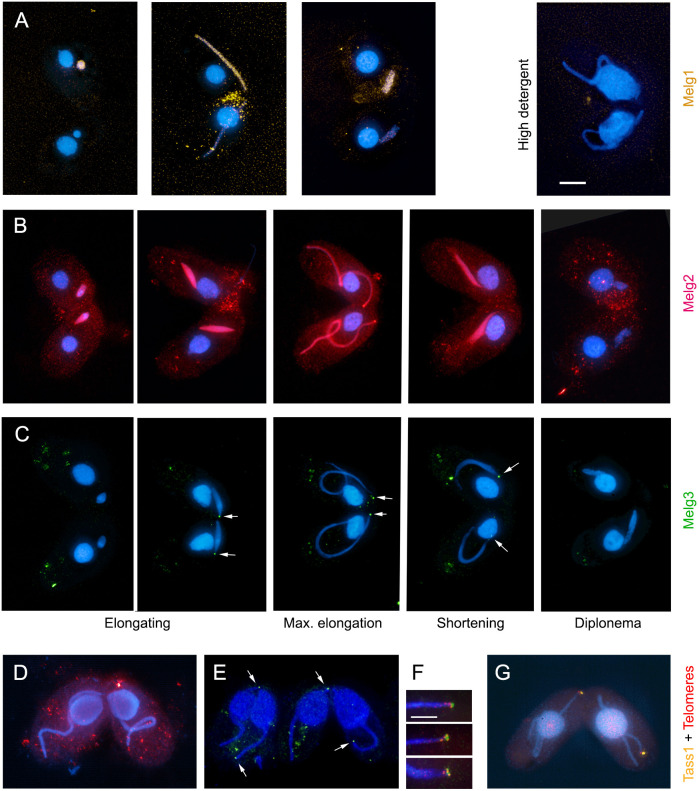
**Localization of Melg proteins.** (A) Melg1-EGFP (orange), overexpressed in one of the partner cells (top), localizes to the meiotic nucleus from the elongating to the shortening stage, and is lost upon high-detergent treatment. (B) Melg2-HA (red) strongly accumulates in the meiotic nucleus from the beginning of elongation to the shortening stage. (C) Melg3-HA (green) localizes to the tip of the meiotic nucleus. Max., maximum. (D) Melg2 is lost upon high-detergent treatment. (E) Melg3 is retained upon high-detergent treatment. (F) Telomeres (red) associate with, but do not fully overlap, the Melg3-decorated tip of the nucleus (green). (G) Tass1 colocalizes with telomeres (signals slightly offset for improved visualization). Arrows in C and E indicate Melg3. Scale bars: 10 µm (A-E,G); 5 µm (F).

In order to study how Melg proteins contribute to meiotic nuclear reorganization, the localization of tagged versions was determined, and interaction partners were identified by co-immunoprecipitation (co-IP), followed by mass spectrometry (MS) (Table S2). GFP-tagged Melg1 could be detected cytologically only upon overexpression. The overexpressed protein was evenly distributed within the meiotic germline nucleus. Its presence was sensitive to high detergent concentrations during cell fixation, suggesting that it localizes to the nucleoplasm (Fig. 5A). Melg1 is a 493-amino-acid protein with leucine-rich repeats (Fig. S3). Inspection of the *Tetrahymena* Genome Database (TGD, www.ciliate.org/, release 2014) identified a clear *MELG1* orthologue in only one of the other three *Tetrahymena* species in TGD, suggesting that it is a rapidly evolving gene. Co-IP experiments, using ectopically expressed EGFP-Melg1 as the bait, identified two partner proteins, Cctδ (encoded by *CCT4*) and Cctη (encoded by *CCT7*) (Table S2). These proteins form part of the conserved multiple-component molecular chaperone CCT, which functions in the assembly of MTs and actin (Stoldt et al., 1996). Two other high-confidence Melg1 partners were TTHERM_00657230p (a homologue of the yeast inner kinetochore protein Skp1) and TTHERM_01266070p (the orthologue of the Rts1 subunit of protein phosphatase 2A, which localizes to the pericentromere in yeast).

Melg2 is a 252-amino-acid protein [encoded by open reading frame (ORF) TTHERM_00289290; NCBI GeneID: 7846875, Uniprot ID, XP_001018646.3] with no conserved domains. HA-tagged Melg2 localized homogenously in the meiotic nucleus (Fig. 5B) during the elongation phase, and its localization was sensitive to high-detergent treatment (Fig. 5D), indicating that it does not bind to chromatin. Melg2 Co-IP experiments identified a glycogen synthase kinase 3 (Gsk3)-like protein (encoded by TTHERM_01016020, NCBI GeneID: 7833181) as a potential partner (Table S2). Gsk3β phosphorylates MT-associated proteins, thereby increasing their binding affinity for MTs and thus MT stability (see Halpain and Dehmelt, 2006). To test whether Gsk3 is involved in nuclear reorganization, we inhibited its activity with LiCl (see Wilson and Lefebvre, 2004). In treated cells, meiotic nuclei did not fully elongate and had a similar appearance to *melg2*Δ nuclei (Fig. S4). However, the cells were ‘sick’, moved slowly and had irregularly shaped somatic nuclei. Nevertheless, the phenocopying effect of lithium suggests that Melg2 may act via Gsk3 to stabilize MTs during meiosis. Melg2 also may have additional functions later in sexual reproduction. Consistent with its MT-related function, anaphase chromosome segregation was disturbed (Fig. S1) and meiotic products were malformed in the *melg2*Δ mutant (Fig. S2). Low levels of Melg2 expression were reported in the precursors of new somatic and germline nuclei after fertilization (Kataoka and Mochizuki, 2015).

Melg3 is a 298-amino-acid protein with no conserved domains (Fig. S3). HA-tagged Melg3 appeared as a small dot at the telomeric tip of the elongated nucleus that did not overlap with telomeres (Fig. 5C,F). Melg3 localization was resistant to high-detergent treatment, indicating that the protein is not soluble in the nucleoplasm (Fig. 5E). As the abundance of endogenous Melg3 was low, overexpressed tagged protein was used to identify partner proteins by co-IP/MS. Notably, three motor proteins (the dyneins Dyh4, Dyh5 and Dyh15) were identified as high-confidence Melg3 partners (Table S2). Two other high-confidence Melg3 partners were Aip1 and Aip2, which were previously shown to interact with the nuclear pore complex subunit Nup85 (the homologue of human Nup107), which is enriched in the germline nucleus (Iwamoto et al., 2017). Two more binding partners were the meiosis-upregulated proteins TTHERM_00637830p and TTHERM_00933110p (Table S2). TTHERM_00637830p-HA could not be detected cytologically and gene knockout retarded vegetative growth (data not shown). TTHERM_00933110p (which we named Tass1; Telomere-associated 1) co-localized with telomere clusters in meiotic prophase nuclei (Fig. 5G). *tass1*Δ mutants have reduced mating efficiency [33% of the cells (*n*=200) were conjugating at 4.5 h after mixing, compared with more than 90% of WT cells]. Co-IP/MS analysis of Tass1 identified Teb1 as a high-confidence interaction partner (Table S2). Teb1 is the DNA-binding subunit of the telomerase holoenzyme (Upton et al., 2014). Together, these results suggest that Melg3 helps to recruit telomeres to the nuclear periphery, and that MT-associated motor proteins are involved in both this process and nuclear elongation.

## DISCUSSION

### Nuclear elongation promotes pairing

*T. thermophila* micronuclei undergo a striking reorganization during meiosis (Fig. 1) – they elongate considerably, and chromosome centromeres and telomeres migrate to opposite tips of the nucleus. Nuclear elongation is proposed to help the pairing of homologous chromosomes by arranging all chromosomes into a bundle of parallel strands in which corresponding regions become juxtaposed (Loidl and Scherthan, 2004; Mochizuki et al., 2008). Nuclear reorganization is disturbed if DSBs are not formed, as in the *spo11*Δ (Mochizuki et al., 2008) or *pars11*Δ mutants (Tian and Loidl, 2018), DSBs are not sensed (upon suppression of the sensor kinase ATR) (Loidl and Mochizuki, 2009) or MT polymerization is inhibited (Kaczanowski et al., 1985; Loidl and Mochizuki, 2009). Under these conditions, either COs are not initiated or meiosis is arrested, which precludes an assessment of the role of nuclear reorganization in meiosis. In contrast, the new *melg* mutants fail to undergo normal nuclear elongation despite DSB formation and sensing, but do progress through all meiotic stages. Hence, these mutants enabled us to confirm that nuclear reorganization in meiotic prophase is important for proper chromosome pairing and recombination (Fig. 4). Surprisingly, reduced nuclear elongation in the *melg1*Δ mutant caused only a moderate reduction in CO and bivalent formation, indicating that nuclear elongation is only one of several factors that contribute to pairing. In fact, centromere and telomere clustering are equally, if not even more, crucial (see below).

### Melg proteins contribute to meiotic nuclear reorganization in different ways

All three meiosis-specific Melg proteins interact with ubiquitous chromosomal and/or cytoskeleton proteins. Therefore, their function is probably to harness these factors in order to reorganize the nucleus in meiotic prophase. Melg1 may achieve this by interacting with the MT-stabilizing factors Cctδ and Cctη. In the absence of Cctδ, MTs have been shown to be gradually lost in *Tetrahymena* (Seixas et al., 2010). Therefore, Melg1 is probably involved in MT assembly and/or stabilization, with loss of Melg1 resulting in incomplete nuclear elongation.

Melg2 may also contribute to MT stabilization, but its more severe mutant phenotype suggests that it has a different functional context. An ultrastructural study (Wolfe et al., 1976) and tubulin immunostaining (Kushida et al., 2015) revealed that intranuclear MTs project from one pole of the nucleus when elongation commences. Simultaneous FISH to telomeres (telFISH) and tubulin staining showed that this was the centromere pole. Thus, nuclear elongation is driven by MT extension from only one end. From its initial monopolar orientation at meiotic prophase, the MT apparatus must reorganize into a bipolar division spindle at metaphase – anaphase I. In the *melg2*Δ mutant, chromosomes precociously entered abnormal anaphase (Fig. 4B; Fig. S1). It is conceivable that Melg2 interacts with Gsk3 to stabilize the monopolar MT apparatus, which retards MT reorganization and anaphase onset. In the absence of Melg2, the precocious initiation of bipolar spindle formation prevents full elongation, proper centromere and telomere attachment and, hence, chromosome pre-alignment. As a consequence, DSBs are repaired via the sister chromatids and univalents segregate.

Melg3 is implicated in the force-generating machinery for elongation. The prevalence of dyneins among the co-IP partners of Melg3 suggests that the forces responsible for nuclear stretching are not predominantly generated by MT polymerization but by MTs sliding along each other. In addition, Melg3 has a function in attaching telomeres to structures at the nuclear tip. This notion is supported by the localization of Melg3 at the telomeric tip of the nucleus, the disruption of telomere clustering in its absence, and its interaction with the telomere-associated protein Tass1 and the nuclear pore complex-interacting proteins Aip1 and Aip2.

Thus, Melg3 is involved in two different aspects of nuclear organization, namely elongation and telomere clustering. Likewise, Melg2 not only functions in elongation but also promotes centromere and telomere clustering at the nuclear tips, and Melg1 (which is not required for centromere clustering) interacts with the putative centromere-related proteins, Skp1-like and Rts1. Thus, regulatory or mechanistic links seem to exist between MT-driven nuclear elongation and centromere and telomere assembly at its ends; however, these await elucidation.

### Centromere and telomere clustering are essential for pairing success

In meiotic prophase of most eukaryotes, chromosome ends attach to the nuclear periphery and are linked to extranuclear motile elements of the cytoskeleton, through which they gain the ability to perform searching movements. They ultimately assemble within a restricted area at the nuclear envelope to form the bouquet. The close vicinity of chromosome axes within the bouquet may promote homologous interactions and/or facilitate the removal of entanglements or heterologous interactions (e.g. Klutstein and Cooper, 2014). Although the telomere cluster in *Tetrahymena* resembles the bouquet, it is believed to result from chromosome shuffling mediated by intranuclear MTs and may, therefore, depend on different structures. In fact, the *Tetrahymena* telomere-associated proteins Melg3 and Tass1 are not homologous to any known bouquet proteins (Lee et al., 2020; Link and Jantsch, 2019), and the lack of bouquet protein homologues in *Tetrahymena* confirms that the bouquet and the telomere cluster of *Tetrahymena* are not homologous devices. Even among plants, fungi and metazoans, the only conserved proteins are SUN and KASH proteins (transmembrane linkers between chromosomes and the motion-generating components of the cytoskeleton), whereas the telomere adaptor proteins that anchor chromosomes to the SUN-KASH bridge are not conserved (da Cruz et al., 2020). This could indicate a polyphyletic origin of the bouquet after the splitting of eukaryotic lineages.

We previously showed that centromere attachment is important for chromosome arms to span between the two ends of the elongated nucleus (Loidl et al., 2012), and our observations suggest that bivalent formation can be quite efficient without telomere association and with limited elongation, as long as centromeres are clustered. Thus, the projection of chromosome arms from the centromere cluster to position homologous regions at similar ‘latitudes’ of the nucleus is sufficient for homology finding. Increasing evidence from other organisms also indicates a role for centromeres in meiotic pairing. Transient (non-homologous) centromere interactions contribute to meiotic pairing in many species (see Corredor et al., 2007; Klutstein and Cooper, 2014; Obeso et al., 2014) and, similar to *Tetrahymena*, the spanning of chromosome arms between a centromere and a telomere cluster has been described in the plant genera *Brachypodium* (Wen et al., 2012), *Zea* (Zhang et al., 2013) and *Triticum* (Sepsi et al., 2017). It is therefore conceivable that centromere clustering derives from interphase centromere co-orientation (the ‘Rabl orientation’), which results from the poleward movement of centromeres in the preceding mitotic anaphase. From this pre-existing order-generating device in pre-meiotic interphase, it is only a small step to homologous centromere association (‘centromere coupling’) and telomere assembly at the opposite pole of the meiotic prophase nucleus. Therefore, we suggest that centromere clustering may have preceded telomere clustering as the primordial organizing principle of meiotic nuclei in early eukaryotes.

## MATERIALS AND METHODS

### Strains and culture conditions

WT *T. thermophila* strains B2086 (mating type II) and CU428 (mating type VII) were obtained from the *Tetrahymena* Stock Center at Cornell University (http://tetrahymena.vet.cornell.edu/). Cells were cultured in Neff's medium at 30°C using standard methods (see Orias et al., 2000), and were made competent for sexual reproduction by starvation in 10 mM Tris-HCl pH 7.4 for 12-16 h. Meiosis was induced by mixing starved cultures of different mating types at equal densities (∼2×10^5^ cells/ml). To inhibit Gsk3 kinase activity (see Wilson and Lefebvre, 2004), 25 mM LiCl was added 2 h after mixing. Cells were fixed 3.5 h and 4.5 h after mixing.

### Somatic gene knockout

For somatic gene knockouts of *MELG1*, *MELG2*, *MELG3* and *TASS1*, ∼50 copies of each target gene in the polyploid somatic macronucleus were replaced with a deletion cassette carrying an antibiotic resistance marker in both mating partners. To generate the plasmid construct for deleting a 1365 bp N-terminal region (including the start codon) from the *MELG1* ORF, a ∼700 bp 5′-UTR fragment and another ∼700 bp ORF fragment were PCR amplified from WT genomic DNA with primer pairs 1–2, and 3–4, respectively (Table S3). Using sequence overlaps, these two fragments and the *MTT1* (metallothionein) promoter plus *CHX* (cycloheximide resistance) cassette, excised from pMcmd1-HA-Chx (Tian and Loidl, 2019) by PstI-XhoI double digestion, were cloned into the SacI-KpnI sites of pBluescript SK(+) by Gibson assembly. For *Tetrahymena* transformation, the *MELG1* knockout construct was linearized by SacI-KpnI double digestion and introduced into starved cells via biolistic transformation (Cassidy-Hanley et al., 1997). Transformants were selected on growth medium containing increasing concentrations of cycloheximide (from 15 to 240 µg/ml) and decreasing concentrations of CdCl_2_ (from 4.5 to 0.05 µg/ml), to gradually replace the ∼50 WT loci in the somatic nucleus with the knockout alleles by way of random assortment. The plasmid construct for deleting the entire *MELG2*, *MELG3* or *TASS1* ORF was generated in the same way, using primer pairs 5–6 and 7–8, 9–10 and 11–12, or 13–14 and 15–16, respectively. As no viable *melg2* transformants were recovered, the CHX cassette was replaced by a *NEO4* (neomycin resistance) cassette (Mochizuki, 2008). Transformants were selected in growth medium with increasing concentrations of the neomycin derivative paromomycin (from 0.12 to 16 mg/ml) and decreasing concentrations of CdCl_2_ (from 1 to 0.1 µg/ml), until complete replacement in the B2086 strain was achieved. As we did not achieve full replacement in the CU428 strain, we removed *MELG2* by the co-deletion method (Hayashi and Mochizuki, 2015). For this, a 1762 bp fragment from the *MELG2* locus (including 786 bp of its ORF) was PCR amplified from genomic DNA with the primer pair 17–18, and cloned into the NotI site of the pMcoDel plasmid (Hayashi and Mochizuki, 2015). For transformation, the intact *MELG2* coDel plasmid was introduced into *Tetrahymena* mating cells via biolistic transformation. Cells were kept in starvation medium overnight, transferred to growth medium and selected with 0.12 mg/ml paromomycin. Viable cells were picked and cultured until they became sexually mature.

Somatic gene knockout candidates were first screened with qPCR using genomic DNA as the template (data not shown), and then absent or significantly reduced mRNA expression from target loci was confirmed by reverse transcription PCR (RT-PCR) or qRT-PCR (Fig. S5A-C). For this, total RNA was extracted using the peqGOLD TriFast solution (PEQLAB) from mating knockout and WT cells at 4 h after induction of meiosis. Next, 1 μg RNA was treated with ezDNase enzyme (Thermo Fisher Scientific) for 2 min and used for cDNA synthesis using the SuperScript IV VILO Master Mix (Thermo Fisher Scientific), according to the manufacturer's instructions. Finally, using cDNA as the template, mRNA expression from the *MELG1*, *MELG2*, *MELG3*, *TASS1* and *TWI1* (loading and stage control) loci was inspected by PCR using primer pairs 19–20, 21–22, 23–24, 25–26, and 27–28, respectively (Fig. S5A,B).

### Protein tagging

For expressing C-terminally HA-tagged Melg2, DNA fragments were PCR amplified from the ORF and 3′ flanking region using primer pairs 29–30 and 31–8, respectively. The fragments were fused to an EGFP-tag sequence and *NEO4*-cassette-containing DNA fragment that had been excised from the pEGFP-*Neo4* plasmid (Kataoka et al., 2010) using BamHI-XhoI double digestion, and then cloned into the SacI-KpnI sites of pBluescript SK(+) using Gibson assembly. Next, the EGFP sequence was excised from the plasmid by BamHI-PstI double digestion and replaced with the HA sequence from the pHA-*NEO4* plasmid (Kataoka et al., 2010) that had been excised using the same restriction enzymes. The plasmid construct for expressing C-terminally HA-tagged Melg3 was generated in the same way but using primer pairs 32–33 and 34–12. These constructs were linearized by SacI-KpnI double digestion and introduced into starved WT cells via biolistic transformation as described above. Transformants were selected in growth medium containing increasing concentrations of paromomycin (from 0.12 to 16 mg/ml) and decreasing concentrations of CdCl_2_ (from 1 to 0.1 µg/ml).

The plasmid used for ectopic expression of N-terminally EGFP-tagged Melg1 was generated using the pBNMB1-EGFP plasmid (a gift from Kazufumi Mochizuki, Dept. Genet. Develop., IGH, CNRS, France). A *MELG1* 5′ flanking sequence and the N-terminal sequence of the ORF were PCR amplified from genomic DNA using primer pairs 1–35 and 19–20, respectively. The fragments were then fused to an EGFP-tag sequence and a *NEO5*-cassette-containing DNA fragment that had been excised from pBNMB1-EGFP using SalI-BamHI double digestion, and then cloned into the SacI-KpnI sites of pBluescript SK(+) using Gibson assembly. The plasmid used for ectopic expression of N-terminally HA-tagged Melg3 was generated using the pBNMB2-HA plasmid (a gift from Kensuke Kataoka, Cell Biol. Dept., Natl. Inst. Basic Biol., Japan). Briefly, the entire *MELG3* ORF was PCR amplified from genomic DNA using the primer pair 36–24, and then cut with BamHI and SpeI. The fragment was then inserted between the BamHI and SpeI sites of the pBNMB2-HA plasmid. The plasmid used for ectopic expression of N-terminally HA-tagged Tass1 was generated in the same way, but using the primer pair 37–26. For transformation, these constructs (or pBNMB1-EGFP) were linearized by SacI-KpnI double digestion and introduced into starved WT cells via biolistic transformation, as described above. Transformants were selected in growth medium containing increasing concentrations of paromomycin (from 0.12 to 2 mg/ml).

For EGFP-Melg1 overexpression, the EGFP-*MELG1* cassette was placed under the control of the *MTT1* promoter, and expression was induced by starving cells in the presence of 0.15 µg/ml CdCl_2_ before meiosis. Overexpression of *HA-MELG3* and *HA-TASS1* was induced in the same way.

### Cell fixation and staining

To analyze meiotic progression, mating cells were fixed at various time points after mixing by adding formaldehyde (final concentration 4%) and Triton X-100 (final concentration 0.5%) to 5 ml of the cell sample. After incubation for 30 min at room temperature, cells were pelleted and resuspended in a solution of 4% paraformaldehyde and 3.4% sucrose. The cell suspension was spread onto a slide and air dried. Slides were then washed twice with 1× PBS and once with 1× PBS containing 0.05% Triton X-100 (PBST). Cell preparations were stained with DAPI in Vectashield anti-fade buffer (Vector Laboratories). The same fixation method was used to immunostain endogenous and tagged proteins. After washing with PBST, primary antibody (anti-HA, H6908, Sigma-Aldrich; anti-Rad51, MS-988, NeoMarkers,; anti-GFP, mouse monoclonal, Takara Bio; anti-mCherry, rabbit polyclonal, Takara Bio; 1:200 dilution, each) was applied and incubated under a coverslip for 1 to 2 h. Slides were then washed and a secondary fluorophore-coupled antibody was applied for 1 h, followed by washing and DAPI staining.

To label centromeric regions, we had a commercial provider raise a rabbit polyclonal antibody against the peptide sequence ARKAYQPKRRSNSNQNQQC of the centromeric histone Cna1 (Cervantes et al., 2006). For Cna1 immunostaining, cells were fixed with Schaudinn's fixative, washed with methanol, and applied as a suspension in methanol to slides (Wenkert and Allis, 1984). Slides were washed twice with 1× PBS, then once with PBST, incubated with anti-Cna1 (1:200 dilution), washed, and then incubated with fluorescence-labelled secondary antibody. After the final washes, slides were mounted with Vectashield containing DAPI.

For Giemsa staining of flattened nuclei, cells were prepared using the method described by Bruns and Brussard (1981). Briefly, cells were fixed with Schaudinn's fixative supplemented with 1% acetic acid, resuspended in a 3:1 mixture of methanol/acetic acid, and applied to a slide. After drying, preparations were hydrolyzed with 5 M HCl (100 µl under a coverslip) for 2 min at room temperature, rinsed in distilled water, and air dried. Slides were stained with 4% Giemsa solution in 1× PBS, dried, and mounted with Euparal (for details see Shodhan et al., 2014).

For telFISH, directly 5′- and 3′-Cy3-labelled (AACCCC)_4_ oligonucleotides were used as a probe. Cell preparations on slides were soaked with water, incubated with 1 M sodium thiocyanate under a coverslip at 90°C for 15 min and then washed twice with 2× saline sodium citrate (SSC), followed by denaturation in 70% formamide in 2× SSC (pH 7.1) for 2 min at 68°C. Slides were then rinsed with ice-cold water and air dried. At the same time, the DNA probe was dissolved, denatured at 95°C for 3 min, and put on ice. An aliquot of 8 μl of the FISH probe in hybridization buffer (50% formamide and 10% dextran sulfate in 2× SSC) was dropped onto a slide and sealed under a coverslip; slides were then incubated in a moist chamber at 37°C for 48 h. After hybridization, slides were washed in hybridization buffer at 37°C for 5 min, followed by 5 min washes in 2× SSC, 1× SSC and 1× PBST. Finally, slides were mounted with Vectashield containing DAPI.

### Microscopy and analysis

Preparations were evaluated using a Zeiss Axioskop fluorescence microscope fitted with appropriate filters. For DAPI-stained and immunostained cells, three-dimensional image stacks of different colour channels were sequentially recorded at 63× magnification with a monochrome SPOT camera (Diagnostic Instruments) using MetaVue software (Universal Imaging), deconvolved using AutoQuant (Media Cybernetics), and projected with ImageJ (http://imagej.nih.gov/ij/). False colours were assigned and merged to composite images using Photoshop (Adobe Systems). Centromere and telomere positions were determined on Cna1-stained and telFISH preparations at 100× magnification. Nuclear lengths were measured on electronic images of Schaudinn-fixed Giemsa-stained preparations using the measuring tool in ImageJ by manual tracing.

### Protein co-immunoprecipitation and mass spectrometry

Cells expressing N-terminally tagged EGFP-Melg1, HA-Melg3, HA-Tass1 or C-terminally tagged Melg2-HA protein were harvested by centrifugation (400 ***g*** for 3 min) 3.5 h after meiotic induction (at maximum nuclear elongation). EGFP-Melg1-expressing cells were resuspended in ice-cold GFP-Trap lysis buffer [150 mM NaCl, 1% Triton X-100, 1 mM PMSF, 1× cOmplete EDTA-free protease inhibitor (Roche)] and homogenized by pipetting. Melg2, Melg3 and Tass1 overexpressing cells were resuspended in ice-cold Tris lysis buffer [30 mM Tris-HCl, 20 mM KCl, 2 mM MgCl_2_, 150 mM NaCl, 0.1% Triton X-100, 1 mM PMSF and 1× cOmplete EDTA-free protease inhibitor (pH 7.4)] and ground in a Dounce homogenizer on ice. Soluble cell lysates from samples and controls were collected and clarified by filtration. In addition, insoluble pellets from HA-Melg3- or HA-Tass1-overexpressing cells and the corresponding controls were extracted with nuclease-containing buffer (1% Triton X-100, 150 mM NaCl and 100 µg/ml benzonase) for 1 h on ice. After nuclease treatment, soluble fractions were harvested by centrifugation (20,000 ***g*** for 10 min), mixed with an equal amount of lysis buffer, and filtered.

EGFP-Melg1-expressing cell lysate was mixed with 25 μl of pre-equilibrated GFP-Trap magnetic agarose beads (ChromoTek) and incubated on a rotor at 4°C for 1 h. Beads were washed four times with 40 volumes of 150 mM NaCl, resuspended in 30 μl of 1× SDS-PAGE loading buffer (125 mM Tris-HCl pH 6.8, 2.3% SDS, 10% glycerol, 10 μg/ml Bromophenol Blue and 5% 2-mercaptoethanol), and boiled for 10 min. HA-Melg3, HA-Tass1 and Melg2-HA lysates were incubated with 200 µl EZview red anti-HA affinity gel (Sigma-Aldrich) for 2 h at 4°C. After washing four times with wash buffer (30 mM Tris-HCl, 20 mM KCl, 2 mM MgCl_2_, 150 mM NaCl, 0.1% Triton X-100 and 1×cOmplete EDTA-free protease inhibitor, pH 7.5) and once with wash buffer without Triton X-100, proteins eluted from the gel were precipitated with 10% trichloroacetic acid (TCA) and boiled in 1× SDS loading buffer. Small aliquots were loaded onto SDS-PAGE gels for western blotting with mouse anti-EGFP monoclonal antibody (1:1000, clone JL8, Clontech) or anti-HA monoclonal antibody (1:1000, clone HA-7, Sigma-Aldrich) to confirm protein precipitation (Fig. S5D).

To detect co-IP partners, TCA-precipitated samples were run on an SDS-PAGE gel for 2 cm, and then protein bands were stained with Coomassie Blue and excised for tryptic digestion. Peptides were separated on an Ultimate 3000 RSLC nano-flow chromatography system and analyzed in a Q Exactive HF Orbitrap mass spectrometer equipped with a Proxeon nanospray source (all from Thermo Fisher Scientific). Raw data were processed and analyzed using MaxQuant and Perseus software packages (Cox and Mann, 2008; Tyanova et al., 2016; for details see Tian and Loidl, 2019).

The SAINTexpress algorithm (v3.6; parameter set to SAINTexpress-spc.exe – L4) was used to identify high-confidence protein-protein interactions (Teo et al., 2014). Potential partner proteins of Melg1, Melg2, Melg3 or Tass1 with a Bayesian false discovery rate of 0.05 or less are listed in Table S2.

## Supplementary Material

Supplementary information

Reviewer comments
