# Supplementary material for: Spatial constraints on chromosomes are instrumental to meiotic pairing
Source: J Cell Sci. 2020 Nov 30;133(22):jcs253724. doi: 10.1242/jcs.253724 (PMC7725606; doi:10.1242/jcs.253724)
Supplement: Supplementary information [file joces-133-253724-s1.pdf]

Fig. S1. *melg* mutant phenotypes. (A) DAPI-stained meiotic time courses of *melg1-3*Δ mutants. In all mutants, the maximum (Max.) length of the elongated germline nucleus is less than the length of the cell, but nuclear shortening and chromosome condensation at diplonema to metaphase I occur normally (compare with the WT in Fig. 1). The *melg1*Δ and *melg3*Δ mutants undergo anaphase I normally, but lagging chromosomes (arrows) are seen in *melg2*Δ. (B,C) DSBs are formed in the *melg1–3*Δ mutants. (B) Dmc1 foci (a marker of DSBs under repair) are present in the *melg* mutants but absent in the *spo11*Δ mutant. (High-detergent treatment prior to fixation removes free Dmc1. Signals in the somatic nuclei are due to cross reaction of the anti-Dmc1 antibody with Rad51.) (C) UV irradiation (UV-C, 254 nm; 50 Joule/m2) does not restore nuclear elongation in the *melg* mutants, but does so in the *spo11*Δ mutant by inducing DNA damage. Bars: 10 µm.

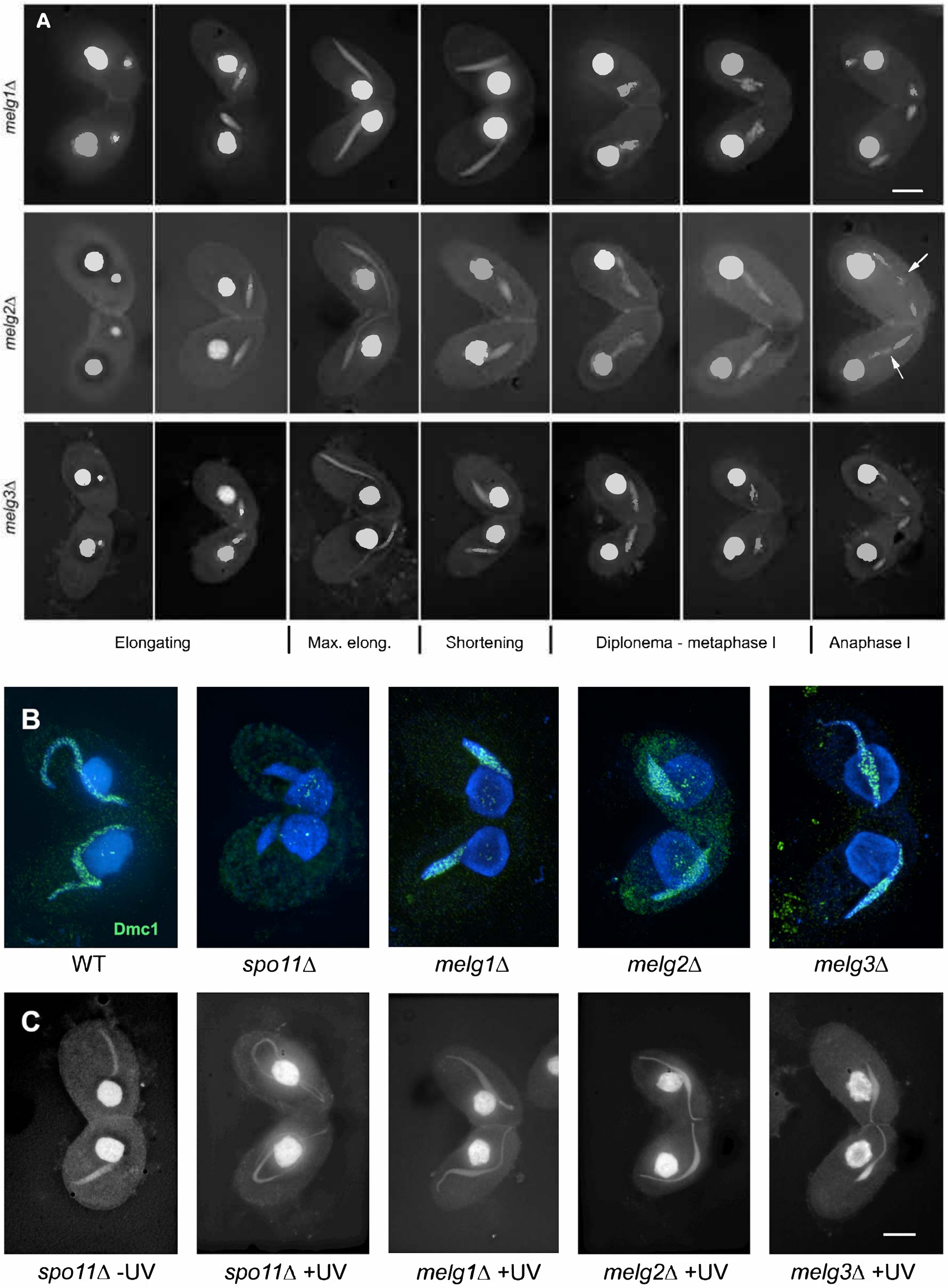

Fig. S2. Late-stage defects in *melg2Δ*. (A) Schematic representation of late meiosis and post meiotic stages in sexual reproduction of WT *Tetrahymena*. For stages up to metaphase I, see Fig. 1A. (B-F) DAPI-stained WT, (G-K) DAPI-stained mutant. (B,G) Interkinesis in the mutant looks similar to that in the WT, except for different nuclear sizes (arrows) due to nondisjoining chromosomes in anaphase I. (C,H,I) Anaphase II is highly disturbed in the mutant compared with the WT. (D,J) In the WT, gametic nuclei (the products of post-meiotic mitosis) migrate to the conjugation junction, pass through membrane pores and fertilize the stationary gametic nucleus of the other cell; in contrast, in the absence of intact gametic nuclei, the somatic nucleus in the mutant becomes abnormally stretched toward the conjugation junction (for comparison, see Akematsu et al., 2020, iScience 23, 100749). (E,F,K) Finally, in the WT, zygotic nuclei (the products of reciprocal fertilization) divide and differentiate into new germline (MIC) and somatic (MAC) nuclei, whereas the old somatic nuclei degenerate. In the mutant, new nuclei do not develop and the old somatic nuclei are retained. Bar: 10 μm.

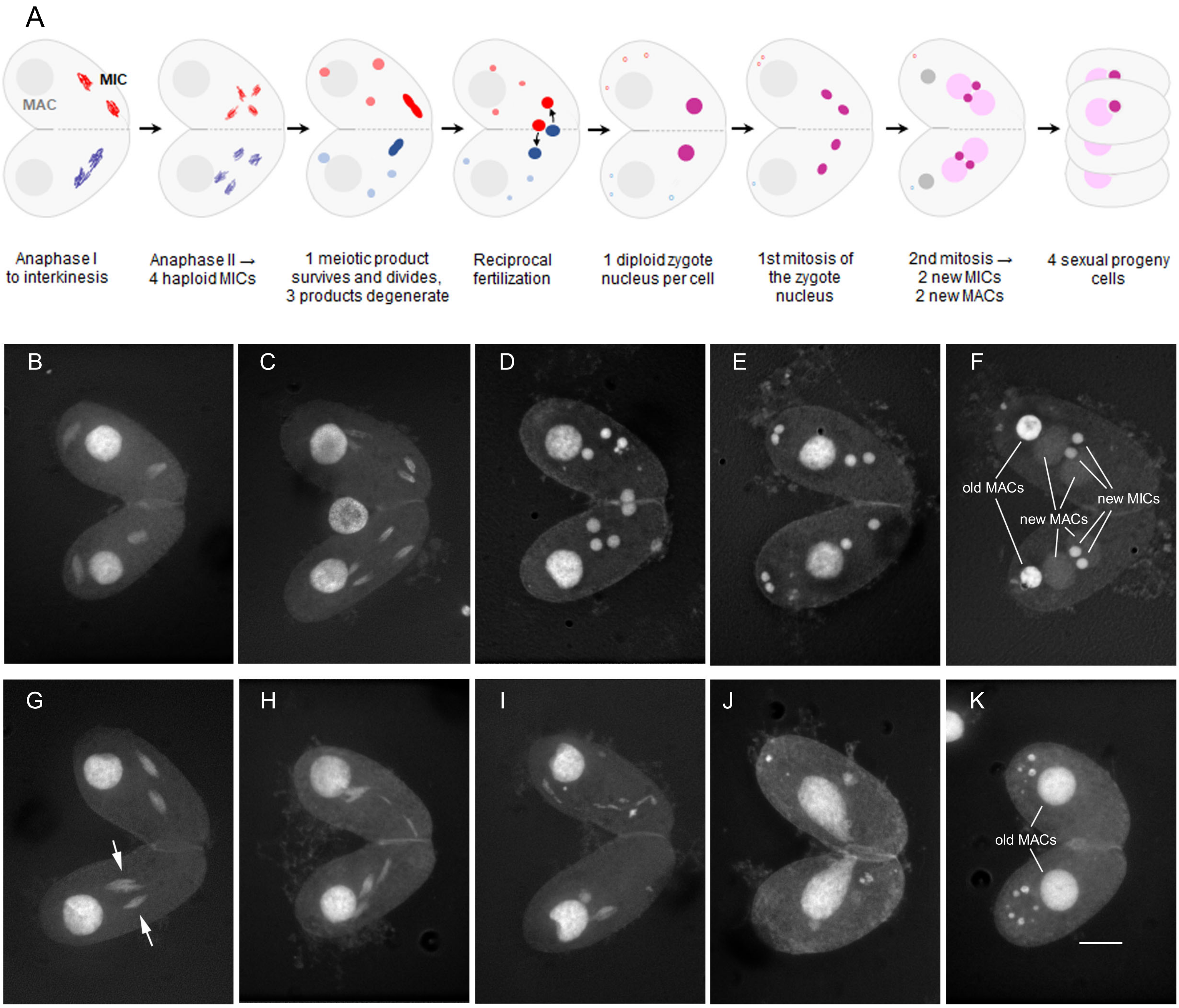

Journal of Cell Science • Supplementary information

Fig. S3

Gene and protein sequences. (A) Melg1 has a ribonuclease inhibitor-like leucine-rich repeats domain (LRR-RI, aa 118 – 261, NCBI BLASTP E-value: 2.73 e-4). This domain is commonly found in ribonuclease inhibitors and Ran GTPase activating proteins (Matsushima et al., 2010, BMC Microbiol. 10, 235). (B) The MELG1 gene has only phase 2 introns and contains multiple 90-bp exon arrays, which are characteristic of young genes created by recent ectopic recombination and/or retrotransposition (Xiong et al. 2019, PLoS Biol. 17, e3000294). (C) MELG3 gene and Melg3 protein sequences. Melg3 is encoded by the gene TTHERM_000365339, which is incorrectly annotated in the current TGD (http://ciliate.org/, release 2020). Based on the transcript sequence (gene_000025916 in TetraFGD (Xiong et al., 2013), we identified an ORF encoding a protein with 298 amino acids.

A

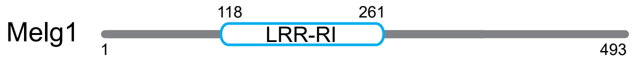

B

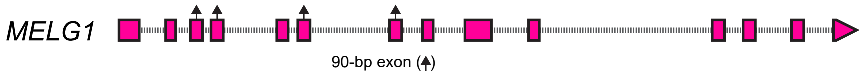

C

>MELG3
ATGGATTAATAGAATATGAGTTTTTCAACAATTGTCTCCTAGATCTCACCTATTTA-
AAGCTAAGATGACACATATAAGAATATCCTTAAGGAAATAGAAAGTAATTACGGAACCAACTCTAATGAAAAAACCAAAAATTAGAACAGTGAAAAA
AAATTACTTCGATAACGCTTTGGAATAATCTATTACATATTAGATGGGTTTTAGTGGCCTATAAGGAATATCAACATTAATACTATGAAAGATATCT
GGTATTAAACTGAGAAATATAAATTTTAAATTATTTAAAAATCTAATCAAAATGATCTTTTACAAAAGATACTCTAAAATGATTTAGAATATGATGA
AATAGCCAAAATAATTATTATCTTAAGTACAACAAGTTAGTCTGATAAAATATATGAAAACTTTGGATTTCTTCTCAATCTTTTGCTAATTGAAAAG
TTAAATTAGCTTAAAAAACAGATTAGCTTGGGGTTCAGAGATGAAAGTCATTCAATCTAAAGTTAATTAAATGTATTTTTATCGAAAATAATTAAAT
CTCTGAAAAATCTAAGCTTACACCTCTACTTTCCCATCCTTATAGAAGATTTAAGGACAGCATTAGAATTTCATCTACATATAATATCTAGCCCATT
GAAAATTATATTTTAAACATCTTTCCAGACGATTGACAATTTGGCAGACATTTTGATTTAGTTCTTCTAACTTTCCTATCCCTTCAAATAAAATGCT
GAAAATATTATCACTTAAGAGCACTTTAACTACTTTTAAGTTCTCATTAAAGAACTTATTACATATCTTTACTTTCCAAAAAATATTTAATCATATG
ACTTATATAATATGGTTAGTTATATCATAAGATAGGTTCTTCCTACTTATTAAAATTTAATATGA

>Melg3
MDQQNMSFSTIVSQISPIQSQDDTYKNILKEIESNYGTNSNEKTKNQNSEKKLLRQRFGII-
YYILDGFQWPIRNININTMKDIWYQTEKYKFQIIQKSNQNDLLQKILQNDLEYDEIAKIIIILSTTSQSDKIYENFGFLLNLLLIEKLNQLKKQISL
GFRDESHSIQSQLNVFLSKIIKSLKNLSLHLYFPILIEDLRTALEFHLHIISSPLKIIFQTSFQTIDNLADILIQFFQLSYPFKQNAENIITQEHFN
YFQVLIKELITYLYFPKNIQSYDLYNMVSYIIRQVLPTYQNLI

Journal of Cell Science • Supplementary information

Fig. S4. Examples of wild type meiotic cells treated with $LiCl_2$.
$LiCl_2$ (25 mM) was added at 2 h after meiotic induction; cells were fixed at 3.5 h after meiotic induction and stained with DAPI. Incompletely elongated meiotic nuclei resemble those of the *melg2*Δ mutant as shown in Fig. 2 and Fig. S1. Bar: 10 µm.

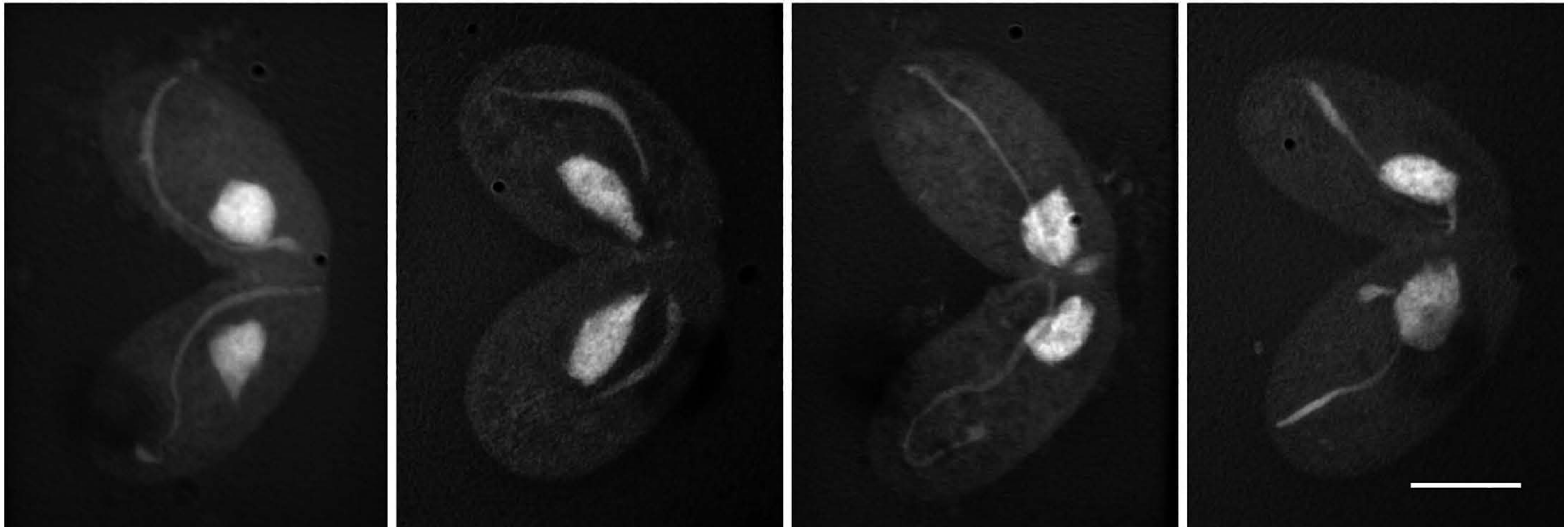

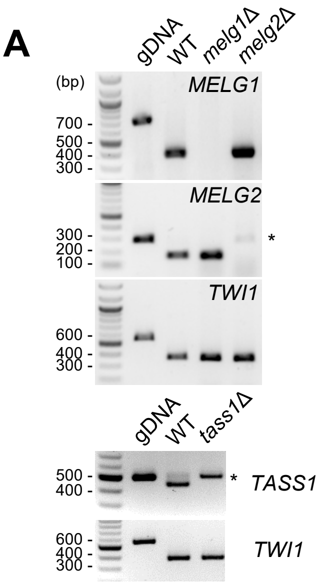

B

Gene	gDNA	cDNA
MELG1	755 bp	425 bp
MELG2	296 bp	178 bp
MELG3	690 bp	690 bp
TASS1	505 bp	445 bp
TWI1	557 bp	372 bp

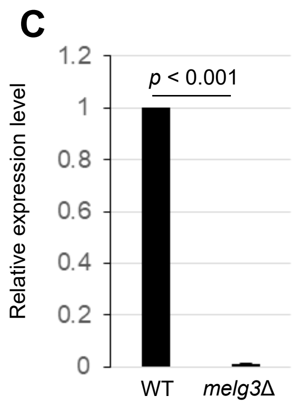

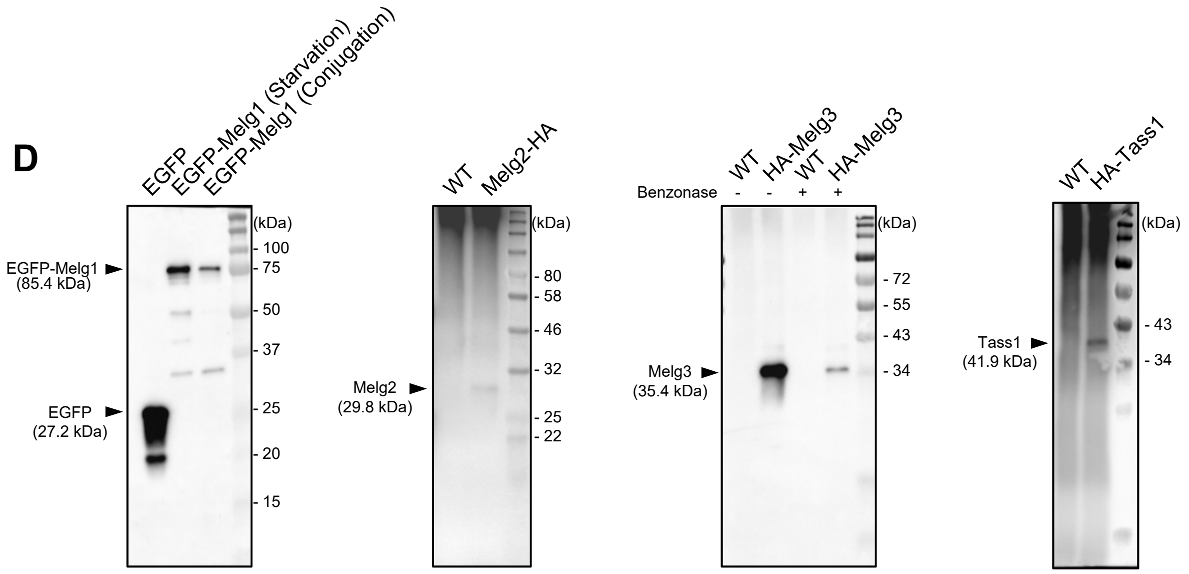

Figure S5. RT-PCR analysis of gene expression in wild-type, *melg1*Δ, *melg2*Δ, *melg3*Δ, and *tass1*Δ cells, and confirmation of IP experiments. (A) Expression of *MELG1*, *MELG2*, *TASS1*, and *TWI1* (control) was investigated by RT-PCR, using cDNA samples generated from total RNAs of wild-type (WT), *melg1*Δ, *melg2*Δ, and *tass1*Δ cells four hours after induction of meiosis. Total RNA samples were treated with DNase before reverse transcription; primer pairs that bind to adjacent exons were used for PCR. PCR products amplified from *Tetrahymena* genomic DNA (gDNA) were used as controls for the size of intron-containing DNA fragments. Asterisks indicate the presence of residual genomic DNA after DNase treatment in *melg2*Δ and *tass1*Δ samples. (B) PCR product sizes. *TWI1* served as a control for the correct staging of samples. (C) For intron-free *MELG3,* the reduction of expression was determined by RT-qPCR. (D) Western blotting of EGFP-Melg1, Melg2-HA, HA-Melg3, and HA-Tass1 IP samples. Proteins were detected with either anti-EGFP or anti-HA antibody.

Journal of Cell Science • Supplementary information

Table S1. Formulas for calculating probabilities of ring bivalents, rod bivalents and univalent pairs. The formulas give approximative probabilities of rings, rods and univalents for a given number of non-interfering crossovers (COs), which occupy chromosome arms randomly. (For simplicity, it is assumed that arm lengths are of equal lengths and probabilities of the two arms of a chromosome are independent. Because of the latter assumption, formulas do not work for low CO numbers.)

X....Number of CO (crossovers)
C....Number of chromosomes (*Tetrahymena*: C=5)
A....Number of Chromosome arms (*Tetrahymena*: A=10)

Explanations	Auxiliary formulas	Probabilities
Probability of each chromosome to get a CO if exactly 1 CO occurs in the cell:<br>Therefore, the probability of a certain chromosome not to obtain a CO is:<br>The probability of not getting a CO (remaining univalent) if X COs occur in the cell is:	P_{hit}=1/C<br><br>$P_{no\ hit}$ =1-1/C	**P_{Univ}= (1-1/C)^X**
The probability of a certain arm of not getting a CO is:<br>The probability of the other arm of the same chromosome not getting a CO is:<br>From this follows the probability of a rod bivalent, namely the probability that one arm gets 0 COs and the other arm does not get 0 COs:<br>(multiplication by 2 because one or the other arm can be affected)	P_{arm} =(1-1/A)^X<br><br>$P_{other\ arm}$ = (1-1/A)^X × (1-(1-1/A)^X)	**P_{Rod}= (1-1/A)^X × 1-(1-1/A)^X × 2**
The probability of a ring bivalent is:		**P_{Ring} =1- P_{Rod}- P_{Univ}**

The observed frequencies of 95.8% rings, 3.7% rods and 0.5% univalents counted in the WT (*n*=400 configurations pooled from (Shodhan et al., 2017) and present counts - Fig. 3 B) are most compatible with an average of 37 COs per cell, calculated to produce 96.0% rings, 4.0% rods and <0.1% univalents. The observed frequencies of configurations for *melg1*Δ *melg2*Δ and *melg*3Δ (Fig. 3 B) fit best with 27, 0.5 and 22 COs per cell, respectively.

Table S2. Melg1, 2, 3 and Tass1 partners identified by co-immunoprecipitation followed by mass spectrometry

Click here to Download Table S2

Journal of Cell Science • Supplementary information

Table S3. List of primers

Primer_ID	Primer name	Primer sequence (5'->3'; adapter sequences for ligation are labelled in red)
#1	Melg1_5UTRf1574_SacG	ACTAAAGGGAACAAAAGCTGGAGCTC ATGAAATGAAAGATATTATCAGAAG
#2	Melg1_5UTRr2244_PstG	ACCCGTCAGGTGCCTGGTACTGCAG TTAATTTAATGGAAACAAGCTATC
#3	Melg1_CDSf3589_XhoG	ACGTCGCACCATGTCGACCTCGAG CAAAAACTATGGAGAGCTTCAC
#4	Melg1_CDSr4249_KpnG	CTCACTATAGGGCGAATTGGGTACC TCTTACACCAGCTTTAGTTATCTAG
#5	Melg2_5UTRf944_SacG	ACTAAAGGGAACAAAAGCTGGAGCTC TTGTCCAAGCATTTATAATACAAC
#6	Melg2_5UTRr1902_PstCG	ACCCGTCAGGTGCCTGGTACTGCAG ACTGCAATCTTGTTAAATATCTAAG
#7	Melg2_3UTRf3038_XhoGK	ACGTCGCACCATGTCGACCTCGAG ATAAATATCTATGAACTCAAAGCAC
#8	Melg2_3UTRr3715_KpnG	CTCACTATAGGGCGAATTGGGTACC ATTCTATTAACTTATCACTAATAGCG
#9	Melg3_5UTRfv1_SacG	ACTAAAGGGAACAAAAGCTGGAGCTC AATCAATCTACTTCTATTTGTTTTG
#10	Melg3_5UTRrv1_PstCG	ACCCGTCAGGTGCCTGGTACTGCAG AACTAAATCATTAATAAGAAATTCC
#11	Melg3_3UTRfv1_XhoGK	ACGTCGCACCATGTCGACCTCGAG ACATGATATTTTATTTAGCAATTAC
#12	Melg3_3UTRrv1_KpnG	CTCACTATAGGGCGAATTGGGTACC ATTAATTAAACAGATTCTTGCTTC
#13	Tass1_5UTRf1004	ACTAAAGGGAACAAAAGCTGGAGCTC AATAACAACTTAAGCCATTCTC
#14	Tass1_5UTRr1670	ACCCGTCAGGTGCCTGGTACTGCAG TTATGGATAGTTTAGTTCATTTTTC
#15	Tass1_3UTRf2892	ACGTCGCACCATGTCGACCTCGAG AGATAGTAGTTTAAATTATACTAAGAAAG
#16	Tass1_3UTRr3630	CTCACTATAGGGCGAATTGGGTACC TTTACTATCAATTAAAGTGAAATTTTAG
#17	Melg2_coDel_fv1	CTTTATTGTTATCATCTTATGACCGC GATGTAGCTCATGAGCTCAG
#18	Melg2_coDel_rv1	CTCATCAAGTTGTAATGCTAAAATGC AATAGAGGAGTGAGGAAGCG
#19	Bamh-Melg1_CDSfv1	TGGTATGGATGAATTATATAAGGGATCC ATGTTGGAATATAACTTAAAGAAATATG
#20	Kpn-Melg1_CDSrv1	CTCACTATAGGGCGAATTGGGTACC AACTTCACTTGTGACTTAATTGTG
#21	Melg2_CDSqf2237	TAGTTTATTCTTATGCATGCTAGAG
#22	Melg2_CDSqr2532	AATCTGATTACCCTAAATCGTAG
#23	Melg3_CDSfv1	ACTAAAGGGAACAAAAGCTGGAGCTC AAAGATATCTGGTATTAAACTGAG
#24	Melg3_NHA_OErv1	CTCAACTAGT TATTAAATTTTAATAAGTAGGAAGAAC
#25	Tass1_Cdsqfv1	TGTTAATGTAGGTTTATCTAAAGTAGC
#26	Tass1_NHAOErv1	CTCAACTAGT TCACTGTTATAAATATTTTTATCTTATTTACATC
#27	Twi1_CDSf3018	TTTAGCAAGTTTATGAATAAGCTC
#28	Twi1_CDSr3574	TAATAGTGTAGTGAGTAGGAGAAGC
#29	Melg2_CDSf2237_SacG	ACTAAAGGGAACAAAAGCTGGAGCTC TAGTTTATTCTTATGCATGCTAGAG
#30	Melg2_CDSr2965_BamG	TCTTCACCCTTAGAAACCATGGATCC ATTATTAAAGTAGCTTTTAGTTGCGTC
#31	Melg2_3UTRf3038_XhoGT	GCTTATCGATACCGTCGACCTCGAG ATAAATATCTATGAACTCAAAGCAC
#32	Melg3_CDSfv1_SacG	ACTAAAGGGAACAAAAGCTGGAGCTC AAAGATATCTGGTATTAAACTGAG
#33	Melg3_CDSrv1_BamG	TCTTCACCCTTAGAAACCATGGATCC TATTAAATTTTAATAAGTAGGAAGAAC
#34	Melg3_3UTRfv1_XhoG	GCTTATCGATACCGTCGACCTCGAG ACATGATATTTTATTTAGCAATTAC
#35	Melg1_5UTRr2244_Sal1n5	TCCATACTTTGAAGATATCAAGTCGAC TTAATTTAATGGAAACAAGCTATC
#36	Melg3_NHA_OEfv1	ATGCTGGATCC ATGGATTAATAGAATATGAGTTTTTC
#37	Tass1_NHAOEfv1	ATGCTGGATCC ATGAACTAAACTATCCATAATATCTATAATAATATG

Journal of Cell Science • Supplementary information
