# Supplementary material for: Spatial constraints on chromosomes are instrumental to meiotic pairing
Source: J Cell Sci. 2020 Nov 30;133(22):jcs253724. doi: 10.1242/jcs.253724 (PMC7725606; doi:10.1242/jcs.253724)
Supplement: Reviewer comments [file joces-133-253724_review_history.pdf]

Spatial constraints on chromosomes are instrumental to meiotic pairing

Miao Tian, Christiane Agreiter and Josef Loidl
DOI: 10.1242/jcs.253724

Editor: David Glover

Review timeline
Original submission: 31 August 2020
Editorial decision: 29 September 2020
First revision received: 7 October 2020
Accepted: 29 October 2020

Original submission

First decision letter

MS ID#: JOCES/2020/253724

MS TITLE: Nuclear spatial constraints are instrumental to meiotic chromosome pairing

AUTHORS: Miao Tian, Christiane Agreiter, and Josef Loidl
ARTICLE TYPE: Research Article

We have now reached a decision on the above manuscript.

To see the reviewers' reports and a copy of this decision letter, please go to: https://submit-jcs.biologists.org and click on the 'Manuscripts with Decisions' queue in the Author Area. (Corresponding author only has access to reviews.)

As you will see, the reviewers gave favourable reports but raised some critical points that will require amendments to your manuscript. I hope that you will be able to carry these out, because I would like to be able to accept your paper.

We are aware that you may currently be unable to access the lab to undertake experimental revisions. If it would be helpful, we encourage you to contact us to discuss your revision in greater detail. Please send us a point-by-point response indicating where you are able to address concerns raised (either experimentally or by changes to the text) and where you will not be able to do so within the normal timeframe of a revision. We will then provide further guidance. Please also note that we are happy to extend revision timeframes as necessary.

Please ensure that you clearly highlight all changes made in the revised manuscript. Please avoid using 'Tracked changes' in Word files as these are lost in PDF conversion.

I should be grateful if you would also provide a point-by-point response detailing how you have dealt with the points raised by the reviewers in the 'Response to Reviewers' box. Please attend to all of the reviewers' comments. If you do not agree with any of their criticisms or suggestions please explain clearly why this is so.

© 2020. Published by The Company of Biologists under the terms of the Creative Commons Attribution License (https://creativecommons.org/licenses/by/4.0/).

Reviewer 1

Advance summary and potential significance to field

This manuscript describes how centromere/telomere clustering and elongation of the nucleus affect pairing of homologous chromosomes in Tetrahymena. Molecular mechanisms for this process of meiosis are diverse among species. Studies in Tetrahymena will provide additional information for understanding the underlying mechanisms in an evolutional view.

Comments for the author

I have some specific comments to improve the manuscript.
1. The title is vague, and should be more specific to the content of the manuscript.

2. Figure S3: Behaviors of the nuclei shown in this figure are too complicated. Thus, it is difficult to understand defects in the melg2-null mutant. Although the process from conjugation to metaphase I is shown in the cartoon in Figure 1, the process shown in Figure S3 occurs after metaphase I, and is not included in the cartoon of Figure 1. A cartoon corresponding to the process of Figure S3 will be helpful for general readers unfamiliar to Tetrahymena.

3. Page 3, first paragraph: How many candidate genes were knocked out to identify three genes?

4. Page 4, first paragraph: The topics of this paragraph is “how defective prophase chromosome arrangements affect homologous pairing”. The concluding sentence appears at the last sentence of the next paragraph “centromere attachment is sufficient to achieve a substantial degree of homologous chromosome prealignment”. These two paragraphs are better to be combined.

5. Figure 4: The lower panel of pictures seems to be a part of panel B, but it’s not obvious. Instead, this part can be a separate panel C. More importantly, it will be helpful to show examples of “ring”, “rod” and “univalent” in the pictures. In the lower panel, the arrows indicate “splayed bivalent ends” as mentioned in the legend to Fig. 4B. The “splayed bivalent ends” are not mentioned in the main text. For these reasons, it is difficult to understand what can be concluded from the presence of “ring”, “rod”, “univalent” and “splayed bivalent ends” in terms of pairing of homologous chromosomes.

6. Citation of literatures seems to be insufficient or biased.
(a) First paragraph in Introduction, “Scherthan 2001”:
This reference is quite old, and it would be better to add more recent reviews, for example:
Zickler D, Kleckner N (2015) Recombination, pairing, and synapsis of homologs during meiosis. Cold Spring Harb Perspect Biol 7:a016626.
Zickler D, Kleckner N. (2016) A few of our favorite things: Pairing, the bouquet, crossover interference and evolution of meiosis. Semin Cell Dev Biol 54:135-148.

(b) First paragraph in Introduction “Loidl 2016”:
This reference is fine, but it may be nice to include some other literatures, for example:
Da Ines O, White CI (2015) Centromere associations in meiotic chromosome pairing. Annu Rev Genet. 49:95-114.

(c) Second paragraph in Introduction: Include references for the statement “Within the elongated nucleus, chromosome arms are arranged side by side, with centromeres and telomeres attached to opposite tips”, for example, Loidl 2012 or some other literatures from the author’s group.

Reviewer 2

Advance summary and potential significance to field

A significant and novel contribution to our understanding of cell biology.

© 2020. Published by The Company of Biologists under the terms of the Creative Commons Attribution License (https://creativecommons.org/licenses/by/4.0/).

This paper explores a mechanism by which meiotic chromosomes align during prophase to permit recombination. The organism, Tetrahymena thermophila, is not a traditional model organism, and therefore allows insight into the evolutionary diversity of meiotic mechanisms. Indeed the molecular machinery involved in attaching centromeres and telomeres to specific loci within the meiotic nucleus appear unique suggesting that ciliates may have invented a chromosome alignment mechanism independently (analogous not homologous) to more conventional mechanisms described in yeast and metazoans. The findings are significant, in that they shed light on the range of mechanisms at work across huge phylogenetic distances to achieve the same result: alignment of homologous chromosomes in anticipation of crossing over. It is novel in that the machinery and mechanisms involved have not been seen before. This paper offers rare and valuable insight into the evolutionary workshop out of which meiotic recombination arose as a mechanism for generating genetic diversity.

Be of broad interest to the cell biology community.
Discovering a novel mechanism behind the process of chromosome alignment is of tremendous interest. As cell biologists, we tend to develop tunnel vision, imagining only the canonical models we are familiar with.
Discovering that ancient organisms invented analogous mechanisms independently speaks to the breadth of mechanistic innovation that evolution has operated with, and highlights the incredibly adaptive value of recombination (selection kept inventing it numerous times using different tools and substrates).

Provide mechanistic insight.
The authors have done a magnificent job using the entire toolbox at their disposal. This has afforded them a great deal of mechanistic detail that is almost all novel and exciting. By mining the data published in the Tetrahymena genome database, they identified a set of genes highly expressed early in meiosis. From these they performed targeted gene knockouts to identify a class of meiotic mutants (ones with abnormally short "crescent" nuclei) for this particular study. The mutant phenotypes were characterized using probes of double-stranded breaks, monitoring the cell's detection of dsbs, localization of centromeres and telomeres (some of the most beautiful fluorescence imaging I've ever seen in ciliates), and even evaluated frequency of crossovers from meiotic karyotypes. It should be noted that not one of these techniques is simple or trivial to perform! Using co-immunoprecipitation followed by Mass Spec, they then identified the potential "partners" that their gene products interact with. Localization data, combined with co-IP allowed the authors to develop a sophisticated set of strongly supported hypotheses regarding the function of these new gene products (no homologs in other model organisms). In one case, the protein they identified by co-IP (Tass1 a telomere binding protein) was also knocked out and its partners identified by co-IP and Mass Spec. The authors are well on their way to unraveling the interactome involved in chromosome alignment during prophase of meiosis I in a truly novel system.

Comments for the author

My only critical remarks, are to point out the rather tenuous nature of conclusions from the GSK3 (LiCl) experiments. Lithium has such broad effects, and the phenocopy was so tenuous, that I would be hesitant to put much weight on these findings.
The authors write: " the phenocopying effect of lithium strongly suggests that Melg2 acts via Gsk3 to stabilize MTs during meiosis." I would argue the conclusion should be more tentative.

Small edits: pp7, 6 lines from the bottom:
therefore, depend on different structures. should read:
therefore, depends on different structures.

I was puzzled by two things in the methods: first, pp9:
Transformants were selected on growth medium containing increasing concentrations of cycloheximide (from 15 to 240 µg/ml) and decreasing concentrations of CdCl2 (from 4.5 to 0.05µg/ml), to gradually replace the 50 WT loci in the somatic nucleus with the knockout alleles...
I understand how CHX was deployed to select for cells with increasing copy number of the disrupted gene locus... but did not understand the role of decreasing the Cadmium. (Was the endogenous locus first replaced with an MTT-driven wildtype allele? that would make sense... but is this

© 2020. Published by The Company of Biologists under the terms of the Creative Commons Attribution License (https://creativecommons.org/licenses/by/4.0/).

described? Maybe I'm missing something.) I was also confused by why CU428 transformants failed to assort. The details of how these knockouts were generated seem a little sketchy.
The title is not ideal. "Nuclear spatial constraints are instrumental to meiotic chromosome pairing". It unfortunately leads the reader to think in terms of the location of the nucleus within the cell cytoplasm.
Tetrahymena nuclei undergo dramatically different fates, meiosis vs programmed nuclear degeneration, vs genome amplification, depending on the spatial localization (spatial constraints) of the relevant nuclei.
Perhaps "Spatial Constraints on Chromosomes are instrumental to meiotic chromosome pairing,", "Intra-nuclear spatial constraints are instrumental to meiotic chromosome pairing." or something that references chromosome alignment.

Reviewer 3

Advance summary and potential significance to field

This work demonstrates that changes in meiotic nuclear morphology are essential for pairing and crossing over in Tetrahymena. It identifies three
novel genes required for these nuclear shape changes, and presents evidence that the effects of mutations in these genes are specific to the process of nuclear elongation rather than the initiation or sensing of double-strand breaks. Importantly, these mutants do not arrest meiosis, but allow assessment of the effects of nuclear elongation defects on pairing and crossing over, and clearly demonstrate a relationship between them, which is a significant finding. Interaction assay strongly hint at roles of these genes in microtubule stability and function in these nuclear shape changes.

Comments for the author
Review of
“Nuclear spatial constraints are instrumental to meiotic chromosome pairing” Miao Tian, Chri stiane Agreiter, and Josef Loidl

Tian et al. describe the identification and characterization of three new genes involved in controlling meiotic nuclear shape changes in Tetrahymena, and use these to explore the relationship between nuclear organization and meiotic pairing and crossing over. Mutations in the three genes each disrupt nuclear elongation, but each differentially affects the process. Importantly, unlike previous mutations in double-stand break induction or sensing, mutants in these genes allow meiosis to proceed and allow observations on pairing and crossing over. The main conclusion that these alterations in nuclear shape play an important role in meiotic pairing is supported by the data.

I found this a very interesting and thorough work. The hypotheses are very clearly presented and the data compelling. There is enough information provided to make the article accessible to those who do not work with this organism. The micrographs are really beautiful and very clearly illustrate each conclusion. Suitable controls are presented for each experiment and the science is very solid. Although the precise functions of these novel genes are not fully elucidated, the authors provide plenty of circumstantial evidence derived from interaction assays to hint at their activities. This work will definitely be of significant interest to both researchers of meiosis and those with more general interest in nuclear structure.

One particularly interesting aspect of their findings is a correlation between the disruption of centromere clustering and the disruption of pairing and crossing over. While this certainly hints at a causative relationship, however, I think the conclusion that they have “demonstrated here that bivalent formation can be quite efficient without telomere association and with limited elongation as long as centromeres are clustered” perhaps overstates their findings. While *melg2* disrupts both centromere clustering and pairing, it is not clear what other aspects of nuclear elongation and organization are disrupted in addition to centromere clustering in this mutant. Thus, it is not clear that the pairing defect can be directly attributed to the defect in centromere clustering. Likewise, although pairing and centromere clustering is retained in the telomere-clustering defective mutants, the conclusion that that cen clustering is requisite for pairing does not follow. Perhaps it would be more accurate to say that “our observations suggest” rather than “demonstrate” here.

© 2020. Published by The Company of Biologists under the terms of the Creative Commons Attribution License (https://creativecommons.org/licenses/by/4.0/).

In a similar vein, although I find it an interesting idea that the role of centromere clustering in pairing may have evolutionarily preceded that of telomere clustering, the data presented here does not really address this. The observation that one mutant that disrupts centromere clustering has a more profound effect on pairing and crossing over than 2 other mutants that disrupt telomere clustering does not really address the temporal order of evolution of these systems.

In addition to these comments, I have one minor suggestion, to include the organism in the title, both for completeness and accuracy
"Nuclear spatial constraints are instrumental to meiotic chromosome pairing in Tetrahymena"

First revision

Author response to reviewers' comments

As requested by the editorial office, the number of supplemental figures was reduced to 5 and the multi-page Table S2 is presented as a separate Excel file.

We thank the reviewers for their helpful comments. We have followed most of their suggestions, as is documented in the following point-by-point response.

Reviewer 1
Q: 1. The title is vague, and should be more specific to the content of the manuscript.
A: DONE! Following suggestion of reviewer 2.

Q: 2. Figure S3: Behaviors of the nuclei shown in this figure are too complicated. Thus, it is difficult to understand defects in the melg2-null mutant. Although the process from conjugation to metaphase I is shown in the cartoon in Figure 1, the process shown in Figure S3 occurs after metaphase I, and is not included in the cartoon of Figure 1. A cartoon corresponding to the process of Figure S3 will be helpful for general readers unfamiliar to Tetrahymena.
A: An explanatory cartoon was included in Fig. S3.

Q: 3. Page 3, first paragraph: How many candidate genes were knocked out to identify three genes?
A: Information added: >80.

Q: 4. Page 4, first paragraph: The topics of this paragraph is "how defective prophase chromosome arrangements affect homologous pairing". The concluding sentence appears at the last sentence of the next paragraph "centromere attachment is sufficient to achieve a substantial degree of homologous chromosome prealignment". These two paragraphs are better to be combined.
A: DONE!

Q: 5. Figure 4: The lower panel of pictures seems to be a part of panel B, but it's not obvious. Instead, this part can be a separate panel C. More importantly, it will be helpful to show examples of "ring", "rod" and "univalent" in the pictures. In the lower panel, the arrows indicate "splayed bivalent ends" as mentioned in the legend to Fig. 4B. The "splayed bivalent ends" are not mentioned in the main text. For these reasons, it is difficult to understand what can be concluded from the presence of "ring", "rod", "univalent" and "splayed bivalent ends" in terms of pairing of homologous chromosomes.
A: DONE! Examples shown in Fig. 4, together with an explanation of the splayed ends.

Q: 6. Citation of literatures seems to be insufficient or biased.
(a)First paragraph in Introduction, "Scherthan 2001": This reference is quite old, and it would be better to add more recent reviews, for example: Zickler D, Kleckner N (2015) Recombination, pairing, and synapsis of homologs during meiosis. Cold Spring Harb Perspect Biol 7:a016626. Zickler D, Kleckner N. (2016) A few of our favorite things: Pairing, the bouquet, crossover interference and evolution of meiosis. Semin Cell Dev Biol 54:135-148. (b)First paragraph in Introduction "Loidl

© 2020. Published by The Company of Biologists under the terms of the Creative Commons Attribution License (https://creativecommons.org/licenses/by/4.0/).

2016”: This reference is fine, but it may be nice to include some other literatures, for example: Da Ines O, White CI (2015) Centromere associations in meiotic chromosome pairing. Annu Rev Genet. 49:95-114. (c) Second paragraph in Introduction: Include references for the statement “Within the elongated nucleus, chromosome arms are arranged side by side, with centromeres and telomeres attached to opposite tips”, for example, Loidl 2012 or some other literatures from the author’s group.
A: References added.

Reviewer 2
Q: My only critical remarks, are to point out the rather tenuous nature of conclusions from the GSK3 (LiCl) experiments. Lithium has such broad effects, and the phenocopy was so tenuous, that I would be hesitant to put much weight on these findings. The authors write: " the phenocopying effect of lithium strongly suggests that Melg2 acts via Gsk3 to stabilize MTs during meiosis." I would argue the conclusion should be more tentative.
A: DONE! Statement weakened.

Q: Small edits: pp7, 6 lines from the bottom: therefore, depend on different structures. should read: therefore, depends on different structures.
A: No. I am more careful here and intend to say that the cluster is believed to depend on..., and not, that it depends on....

Q: I was puzzled by two things in the methods: first, pp9: Transformants were selected on growth medium containing increasing concentrations of cycloheximide (from 15 to 240 μg/ml) and decreasing concentrations of $CdCl_2$ (from 4.5 to 0.05μg/ml), to gradually replace the 50 WT loci in the somatic nucleus with the knockout alleles... I understand how CHX was deployed to select for cells with increasing copy number of the disrupted gene locus... but did not understand the role of decreasing the Cadmium. (Was the endogenous locus first replaced with an MTT-driven wildtype allele? that would make sense... but is this described? Maybe I'm missing something.) I was also confused by why CU428 transformants failed to assort. The details of how these knockouts were generated seem a little sketchy.
A: We occasionally have the problem that we raise the selecting antibiotics concentration (to as high as 40 mg/ml in the case of PM) and cells are resistant without being fully assorted as tested by qPCR. This is also the reason why we decrease $CdCl_2$ concentrations. The rationale is that weaker stimulation of antibiotic resistance by lower conc of Cd is only sufficient for those cells that have higher copy numbers of the resistance cassette.

Q: The title is not ideal. "Nuclear spatial constraints are instrumental to meiotic chromosome pairing". It unfortunately leads the reader to think in terms of the location of the nucleus within the cell cytoplasm. ... Perhaps "Spatial Constraints on Chromosomes are instrumental to meiotic chromosome pairing,", "Intra-nuclear spatial constraints are instrumental to meiotic chromosome pairing." or something that references chromosome alignment.
A: DONE!

Reviewer 3
Q: One particularly interesting aspect of their findings is a correlation between the disruption of centromere clustering and the disruption of pairing and crossing over. While this certainly hints at a causative relationship, however, I think the conclusion that they have “demonstrated here that bivalent formation can be quite efficient without telomere association and with limited elongation as long as centromeres are clustered” perhaps overstates their findings. While melg2 disrupts both centromere clustering and pairing, it is not clear what other aspects of nuclear elongation and organization are disrupted in addition to centromere clustering in this mutant. Thus, it is not clear that the pairing defect can be directly attributed to the defect in centromere clustering. Likewise, although pairing and centromere clustering is retained in the telomere-clustering defective mutants, the conclusion that that cen clustering is requisite for pairing does not follow. Perhaps it would be more accurate to say that “our observations suggest” rather than “demonstrate” here.
A: DONE!

Q: In a similar vein, although I find it an interesting idea that the role of centromere clustering in pairing may have evolutionarily preceded that of telomere clustering, the data presented here does not really address this. The observation that one mutant that disrupts centromere clustering has a

© 2020. Published by The Company of Biologists under the terms of the Creative Commons Attribution License (https://creativecommons.org/licenses/by/4.0/).

more profound effect on pairing and crossing over than 2 other mutants that disrupt telomere clustering does not really address the temporal order of evolution of these systems.
A: The proposition was toned down to a speculation.

Q: In addition to these comments, I have one minor suggestion, to include the organism in the title, both for completeness and accuracy “Nuclear spatial constraints are instrumental to meiotic chromosome pairing in Tetrahymena”.
A: I have changed the title following the suggestion of reviewer 2. I preferred not to include the organism in order not to daunt general readers before they even have a look at the abstract.

Second decision letter

MS ID#: JOCES/2020/253724

MS TITLE: Spatial constraints on chromosomes are instrumental to meiotic chromosome pairing

AUTHORS: Miao Tian, Christiane Agreiter, and Josef Loidl
ARTICLE TYPE: Research Article

I am happy to tell you that your manuscript has been accepted for publication in Journal of Cell Science, pending standard ethics checks.

© 2020. Published by The Company of Biologists under the terms of the Creative Commons Attribution License (https://creativecommons.org/licenses/by/4.0/).
